# Update in Systemic and Targeted Therapies in Gastrointestinal Oncology

**DOI:** 10.3390/biomedicines6010034

**Published:** 2018-03-16

**Authors:** Nelson S. Yee

**Affiliations:** Division of Hematology-Oncology, Department of Medicine, Penn State Health Milton S. Hershey Medical Center, Experimental Therapeutics Program, Penn State Cancer Institute, The Pennsylvania State University College of Medicine, Hershey, PA 17033, USA; nyee@pennstatehealth.psu.edu; Tel.: +1-717-531-0003

**Keywords:** biliary tract carcinoma, chemotherapy, clinical trial, colorectal carcinoma, gastric carcinoma, gastrointestinal oncology, hepatocellular carcinoma, immunotherapy, pancreatic carcinoma, targeted therapy

## Abstract

Progress has been made in the treatment of gastrointestinal cancers through advances in systemic therapies, surgical interventions, and radiation therapy. At the Multi-Disciplinary Patient Care in Gastrointestinal Oncology conference, the faculty members of the Penn State Health Milton S. Hershey Medical Center presented a variety of topics that focused on this sub-specialty. This conference paper highlights the new development in systemic treatment of various malignant diseases in the digestive system. Results of the recent clinical trials that investigated the clinical efficacy of pegylated hyaluronidase, napabucasin, and L-asparaginase in pancreatic carcinoma are presented. The use of peri-operative chemotherapy comprised of 5-fluorouracil or capecitabine, leucovorin, oxaliplatin, and docetaxel (FLOT), and immunotherapy including pembrolizumab, nivolumab, and ipilimumab in gastroesophageal carcinoma are discussed. Data from clinical trials that investigated the targeted therapeutics including nivolumab, ramucirumab, lenvatinib, and BLU-554 are reported. The role of adjuvant capecitabine in resected biliary tract carcinoma (BTC) and nab-paclitaxel in combination with gemcitabine and cisplatin in advanced BTC are presented. In colorectal carcinoma, the efficacy of nivolumab, adjuvant FOLFOX or CAPOX, irinotecan/cetuximab/vemurafenib, and trifluridine/tipiracil/bevacizumab, is examined. In summary, some of the above systemic therapies have become or are expected to become new standard of care, while the others demonstrate the potential of becoming new treatment options.

## 1. Introduction

Cancers in the digestive system are among the most common malignant diseases worldwide, and are associated with relatively high mortality rates. Optimal caring of patients with malignant diseases of digestive organs requires the expertise of providers from multiple health disciplines. At the Multi-Disciplinary Patient Care in Gastrointestinal Oncology conference held on 29 September 2017 in Hershey, Pennsylvania, the faculty members of the Penn State Cancer Institute and Penn State Health Milton S. Hershey Medical Center presented a variety of topics that focused on this sub-specialty. These included presentations regarding the new frontiers in diagnostic and staging evaluation, surgical interventions, and radiation therapy. In particular, important advances in systemic chemotherapy, as well as the discovery of molecular biomarkers and therapeutic targets in those cancers, were discussed. This article focuses on the presentations about new development of systemic and targeted therapies in various malignancies of the digestive system.

## 2. Update of Systemic and Targeted Therapies

A number of advances have been made in the systemic and targeted treatment of cancers of the digestive organs. These include cancers in the pancreas, stomach and gastroesophageal junction, liver, biliary tract, colon, and rectum. Here is a summary of the most recent evidence on chemotherapy and targeted therapy in gastrointestinal oncology as presented at the international medical conferences and in the published medical literature. Those conferences were held in 2017, including Annual Conference of the American Society of Clinical Oncology, Annual Conference of the European Society of Medical Oncology World Gastrointestinal Congress, and the Annual Conference of the International Liver Cancer Association in Seoul, South Korea.

## 3. Pancreatic Carcinoma

Systemic chemotherapy plays important roles in the treatment of pancreatic carcinoma diagnosed at all stages [1]. New development has been focusing on targeted agents directed against the tumor microenvironment, cancer stem cells, and cellular metabolism [2]. In this section, highlights of the recent clinical trials investigating the clinical efficacy of (i) hyaluronidase that degrades tumor-associated stroma, (ii) napabucasin that targets cancer stem cells, and (iii) l-asparaginase that inhibits cellular metabolism, are reported.

### 3.1. Targeting Tumor-Associated Stroma Using Pegylated Hyaluronidase

A unique feature of pancreatic carcinoma is desmoplastic reaction, resulting in formation of a dense stroma that hinders delivery of therapeutic agents to the cancer cells. Hyaluronan is a component of the tumor-associated stroma, and pegylated hyaluronidase (PEGPH20) has been developed to degrade hyaluronan in order to facilitate delivery of chemotherapeutic agents to the cancer cells. In a phase II, open-label clinical study of 246 patients with metastatic pancreatic adenocarcinoma, nab-paclitaxel (125 mg/m^2^ iv weekly for 3 weeks) + gemcitabine (1000 mg/m^2^ iv weekly for 3 weeks) with or without PEGPH20 (3 µg/kg iv twice a week for cycle 1 and weekly for cycle 2 and beyond) were administered for every 4-weeks cycle (ClinicalTrials.gov identifier: NCT01839487). PEGPH20 in combination with nab-paclitaxel and gemcitabine significantly prolongs progression-free survival (PFS), as compared to nab-paclitaxel and gemcitabine (Table 1). The improvement of PFS is even greater for patients with tumors expressing high levels of HA, and this is also seen with overall survival (OS). Moreover, this study suggested that hyaluronic acid is a potential predictive biomarker of tumor response to PEGPH20 [3]. A phase III, randomized, double-blind, placebo-controlled, multicenter clinical study is ongoing to confirm the clinical efficacy of PEGPH20 in combination with nab-paclitaxel and gemcitabine (NCT02715804).

### 3.2. Targeting Cancer Stem Cells by a STAT3 Inhibitor

Tumor metastasis and therapeutic resistance have been attributed to cancer stem cells. Targeting the cancer stem cells is expected to improve survival through improving anti-tumor response to treatment. A first-in-human clinical trial was conducted to investigate the clinical efficacy of napabucasin that targets cancer stem cells by inhibiting the activation of signal transducer and activator of transcription 3 (STAT3). In a phase 1b/II study of 66 patients (55 evaluable) with metastatic pancreatic adenocarcinoma, napabucasin 240 mg orally twice daily in combination with nab-paclitaxel (125 mg/m^2^ iv) and gemcitabine (1000 mg/m^2^ iv) was administered weekly for 3 weeks of every 4-week cycle until disease progression (NCT02231723). Results of this study indicate that the combination of napabucasin with nab-paclitaxel and gemcitabine produced anti-tumor response (Table 2) [4]. A phase III, open-label clinical study to confirm the efficacy of napabucasin in combination with nab-paclitaxel and gemcitabine in patients with metastatic pancreatic adenocarcinoma is ongoing (NCT02993731).

### 3.3. Targeting Asparagine by Enzymatic Degradation

Asparagine is an essential amino acid required for survival of pancreatic cancer cells, which have no or little asparagine synthetase to produce endogenous asparagine. Asparagine promotes proliferation of cancer cells through regulating serine uptake, influencing serine metabolism and thus synthesis of protein and nucleotides [5]. Eryaspase consists of L-asparaginase encapsulated within erythrocytes, and it hydrolyzes and depletes asparagine from the circulating blood plasma (http://erytech.com/ery-asp.html). In a phase IIb study, 140 patients with metastatic pancreatic carcinoma were randomized to receive eryaspase (100 IU/kg on day 3 and day 17 of every 4-weeks cycle) in combination with either gemcitabine or FOLFOX (5-fluorouracil, leucovorin, and oxaliplatin); versus chemotherapy alone (NCT02195180). Expression of asparagine synthetase was either none (0) or low (1) as determined by immunohistochemistry. Eryaspase in combination with either gemcitabine or FOLFOX significantly prolonged overall survival as compared to either gemcitabine or FOLFOX (26.1 weeks vs 19 weeks, HR 0.57, * *P* = 0.03) [6].

While some of the above clinical trials are ongoing, the evidence suggests that subsets of patients whose tumors with expression of certain molecular biomarkers likely benefit from the targeted therapeutic agents. Conceivably, patients with pancreatic carcinoma with high expression level of HA may benefit from addition of PEGPH20 to either nab-paclitaxel/gemcitabine or FOLFIRINOX. Those with pancreatic cancer cells with constitutively activated STAT3 may benefit from addition of napabucasin to nab-paclitaxel/gemcitabine. Pancreatic carcinoma with no or low level of expression of asparagine synthetase will likely be sensitive to treatment with eryaspase in combination with either gemcitabine or FOLFOX.

## 4. Gastric, Gastroesophageal Junction, and Esophageal Carcinoma

Systemic chemotherapy is the standard of care for patients with advanced or metastatic gastric carcinoma (GC), gastroesophageal junction carcinoma (GEJC), and esophageal carcinoma (EC). Monoclonal antibodies including trastuzumab directed against human epidermal growth factor receptor 2 (HER2) and ramucirumab that targets vascular endothelial growth receptor 2 (VEGFR2), either as single agents or in combination with chemotherapy, have become part of the standard of care for treatment of advanced GC and GEJC. For resectable GC or GEJC, chemotherapy improves the surgical outcome; whereas chemotherapy concurrent with radiation therapy with or without surgery can be potentially curable for localized EC. Immunotherapy has emerged as a new treatment option for patients with gastroesophageal carcinoma [7]. In this section, highlights of the clinical studies that investigated peri-operative chemotherapy and immunotherapy are discussed.

### 4.1. Peri-Operative FLOT in Resectable Tumors

For resectable GC or GEJC, peri-operative chemotherapy using epirubicin, cisplatin and either 5-fluorouracil or capecitabine (ECF and ECX, respectively) has been the standard of care. However, the survival benefit of this regimen remains limited. The combination of docetaxel, oxaliplatin, and either 5-fluorouracil or capecitabine (FLOT) produced survival benefit in advanced gastroesophageal carcinoma, but its clinical efficacy in resectable tumors had not been determined. In a phase III trial (FLOT4) of 716 patients with resectable GC or GEJC, peri-operative FLOT was compared with ECF or ECX. In the treatment group receiving FLOT, 4 pre-operative and 4 post-operative 2-week cycles of docetaxel 50 mg/m^2^ iv, oxaliplatin 85 mg/m^2^ iv, leucovorin 200 mg/m^2^ iv, and 5-fluorouracil 2600 mg/m^2^ as 24h iv infusion, all were administrated on day 1. For the ECF or ECX treatment group, the patients received 3 pre-operative and 3 post-operative 3-week cycles of epirubicin 50 mg/m^2^ iv and cisplatin 60 mg/m^2^ iv both on day 1, and either 5-fluorouracil 200 mg/m^2^/24 h continuous iv infusion or capecitabine 1250 mg/m^2^ orally on day 1 through day 21 (NCT01216644). Results of this study showed that, as compared to either ECF or ECX, FLOT significantly prolonged PFS and OS, associated with lower rate of disease progression, and increased rate of complete resection of tumors (Table 3). ECF or ECX was associated with more grade 3 or 4 nausea and emesis than FLOT, which was associated with more grade 3 or 4 neutropenia [8]. This study supports peri-operative FLOT as the new standard of systemic treatment for patients with resectable GC or GEJC.

### 4.2. Pembrolizumab in PD-L1-Expressing Gastric and Gastroesophageal Carcinoma

In a phase I clinical study, the anti-programmed death-1 (anti-PD-1) antibody pembrolizumab exhibited anti-tumor activity in patients with previously treated advanced GC. In a phase II clinical study, 259 patients with recurrent or metastatic GC or GCJC that have progressed on 2 or more prior chemotherapy received pembrolizumab (200 mg iv every 21 days) (KEYNOTE-059, NCT02335411). Pembrolizumab significantly improved response rate and overall survival beyond 2nd line treatment in patients with advanced GC or GEJC expressing PD-L1 (≥1% expression of PD-L1). The overall response rate (ORR) is 16.4% in all patients. Among patients with ≥1% expression of PD-L1, ORR 22.7%, vs. 8.6% if PD-L1 negative [9]. Results of this study has led to US FDA approval of pembrolizumab for treatment of patients with advanced, PD-L1-positive (≥1%) GC or GEJC that have progressed following 2 or more lines of therapy.

### 4.3. Nivolumab and Ipilimumab in Gastric, Gastroesophageal Junction, and Esophageal Carcinoma

A combination of immune checkpoint inhibitors was investigated in advanced GC, GEJC, and EC. In a previous phase III clinical study (ONO-12), the anti-PD-1 antibody nivolumab has been shown to prolong overall survival (OS), as compared to placebo, as a 3rd-line or beyond treatment in Asian patients with advanced GC or GEJC [10]. In a phase I/II study, nivolumab in combination with or without the anti-CTLA4 antibody, ipilimumab, produced anti-tumor activity in Western patients with advanced GC, GEJC, and EC (CheckMate 032, NCT01928394). The data of a long-term follow-up of the CheckMate 032 study were reported [11]. In the phase II study, nivolumab either alone or in combination with ipilimumab showed durable anti-tumor responses in metastatic GC or GEJC, particularly in tumors expressing PD-L1 (≥1%), and also long-term OS in those Western patients with previously treated GC, GEJC, and EC (Table 4).

### 4.4. Pembrolizumab in PD-L1-Expressing Esophageal Carcinoma

For patients with advanced or metastatic EC, the conventionally used systemic chemotherapy has been extrapolated from evidence of clinical studies in GC or GEJC. In a phase IB study, pembrolizumab was investigated in patients with PD-L1-positive advanced esophageal carcinoma (KEYNOTE-028, NCT02054806). Among the 83 patients being evaluated, 37 (45%) had PD-L1-positive tumors and 23 patients were enrolled. ORR was 30% (95% CI, 13% to 53%), and median duration of response 15 months (6 to 26 months). Treatment-related adverse events were reported in 9 patients, and those most commonly include anorexia, lymphocytopenia, and skin rash. There were no grade 4 pembrolizumab-related adverse events or deaths. Increased tumor response and delayed tumor progression were associated with relatively high interferon-γ composite scores. It was concluded that pembrolizumab produced durable anti-tumor activity and manageable toxicity in patients with pretreated, PD-L1-positive advanced esophageal carcinoma [12].

The promising data of the above mentioned clinical studies are expected to create new opportunities of effective treatment for patients with GC, GEJC, and EC. While pembrolizumab has been FDA-approved as a 3rd-line or beyond treatment of advanced GC and GEJC expressing PD-L1, the potential use of pembrolizumab either alone or in combination with cytotoxic chemotherapy is being explored as 1st-line treatment. Meanwhile, nivolumab, either as a single agent or in combination with ipilimumab, may provide additional treatment options for patients with GC, GEJC, and EC. The use of peri-operative FLOT is expected to be the new standard of care for patients with resectable GC and GEJC. However, this regimen tends to be associated with severe neutropenia, such that precaution will need to be taken for patients with impaired bone marrow reserve, and granulocyte-colony stimulating factors may need to be routinely administered as part of this regimen.

## 5. Hepatocellular Carcinoma

For patients with advanced or metastatic hepatocellular carcinoma (HCC), sorafenib is the standard first-line treatment. For HCC that has progressed following treatment with sorafenib, regorafenib as a second-line treatment provides survival benefit. However, treatment using sorafenib or regorafenib is palliative, and the clinical benefits of these targeted agents are somewhat limited [13]. Results of clinical trials that investigated the clinical efficacy of targeted therapeutic agents including nivolumab, ramucirumab, lenvatinib, and BLU-554 have recently been reported.

### 5.1. Nivolumab

The efficacy of nivolumab, an anti-PD-1 antibody, was investigated in sorafenib naïve or sorafenib-treated patients with advanced HCC. In this clinical study (CheckMate 040), 262 patients were enrolled and 98% of them had hepatic cirrhosis of Child-Pugh scores 5–6. In both groups of subjects, those who had never received sorafenib and those who had previously been treated with sorafenib, nivolumab showed anti-tumor response to varying extent (Table 5). Importantly, nivolumab produced tumor responses regardless of the etiology of HCC or tumor expression of PD-L1. Based on results of this study, FDA approved nivolumab for treatment of patients with HCC following prior sorafenib regardless of PD-L1 status [14].

In the CheckMate-040 study, the efficacy of nivolumab was further evaluated in a subgroup of 154 patients with HCC who progressed on sorafenib or who were intolerant to sorafenib with additional eligibility criteria (NCT01658878). By assessment using RECIST v1.1, the overall response rate (ORR) was 14.3%, complete response (CR) 1.9%, and partial response (PR) 12.3%. Among those who responded to nivolumab, 91% of the patients had a response duration ≥6 months, and 55% with duration ≥12 months. Assessment using modified RECIST criteria, the efficacy of nivolumab is even greater, with ORR 18.2%, CR 3.2%, and PR 14.9%.

However, nivolumab is associated with the risk of various immune-related adverse reactions. Skin rash is relatively common. Other reactions include pneumonitis, colitis, hepatitis, endocrinopathies (in hypophysis, adrenal, thyroid, endocrine pancreas), nephritis, encephalitis, and infusion-related. These reactions may require treatment with corticosteroids. Depending on the severity of the reactions, nivolumab may need to be withheld or discontinued.

### 5.2. Ramucirumab

Hepatocellular carcinogenesis is promoted by tumor-associated neo-angiogenesis, and vascular endothelial growth factor (VEGF) and receptor-induced signaling plays an important role [13]. The anti-VEGF antibody bevacizumab was previously investigated in advanced HCC without proven clinical benefit. Ramucirumab is a monoclonal antibody directed against VEGFR2 and it produced anti-tumor response in colon, gastric and gastroesophageal junction, and pulmonary carcinoma. The anti-tumor effect of ramucirumab in HCC was previously known.

In a phase III trial (REACH), the clinical efficacy of ramucirumab was investigated in patients with advanced HCC. In this study, 643 patients with advanced HCC who had previously been treated with sorafenib, were randomized to receive ramucirumab or placebo. Results of this study showed that ramucirumab produced statistically significant improvement of the hazard ratio of HCC in patients with hepatic cirrhosis of Child-Pugh 5. The efficacy is further increased in patients with hepatic cirrhosis of Child-Pugh 5 and serum AFP ≥ 400 ng/mL (Table 6). Thus, ramucirumab produced survival benefit in patients with advanced HCC, particularly those with hepatic cirrhosis of Child-Pugh Class A (score 5 or 6) and serum AFP ≥ 400 ng/mL [15].

### 5.3. Lenvatinib

Lenvatinib is a small molecule inhibitor of receptor tyrosine kinases (RTKs) involved in neo-angiogenesis by inhibiting the kinase activities of VEGFR1, 2, and 3. Lenvatinib also inhibits RTKs implicated in tumor growth and metastasis, including fibroblast growth factor receptors (FGFR1, 2, 3, and 4); platelet-derived growth factor receptor (PDGFRα), KIT, and RET. Currently, lenvatinib is FDA-approved for treatment of patients with locally recurrent or metastatic, progressive, radioactive iodine-refractory differentiated thyroid cancer (available online: https://www.accessdata.fda.gov/drugsatfda_docs/label/2015/206947s000lbl.pdf). In a phase III trial, the clinical efficacy of lenvatinib versus sorafenib was investigated as 1st-line treatment of unresectable HCC. In this open-label study, 954 subjects with advanced HCC and hepatic cirrhosis of Child-Pugh A were randomized to receive either lenvatinib or sorafenib as first-line therapy [16]. Results of this study show that lenvatinib produced significant improvements in PFS, TTP, and ORR (Table 7). The investigators concluded that lenvatinib is non-inferior to sorafenib in overall survival.

### 5.4. BLU-554

FGF19 stimulates proliferation of hepatocytes and induces hepatocellular carcinoma through activation of FGFR4 [17]. BLU-554 is a highly selective small molecular inhibitor of FGFR4, and FGF19 has been identified as a potential predictive biomarker of treatment response to BLU-554. A phase I clinical trial was conducted to investigate the safety and efficacy of BLU-554 in patients with advanced HCC that had been pre-treated (Table 8). This trial includes a dose-escalation phase and an expansion portion. Of the first 77 enrolled subjects being analyzed, 44 of them had FGF19-expressing (at least 1% expression) HCC. In the dose-escalation phase, the maximum-tolerated dose of 600 mg of BLU-554 daily was established. BLU-554 produced anti-tumor response in FGF19+ HCC. Patients with HCC without expression of FGF19 did not demonstrate anti-tumor response to BLU-554. [18]. This study suggests the potential use of BLU-544 as a new treatment of patients with HCC that express FGF19.

While nivolumab produces efficacy in a subset of patients with advanced HCC previously treated with sorafenib, future investigation is indicated to improve the response rate conceivably by combination of nivolumab with other interventions. These may include radiation therapy, other immune checkpoint inhibitors, or chemotherapy. The clinical data showing the efficacy of ramucirumab in patients with HCC and elevated serum AFP levels appear promising. It will be worthy to determine if a combination of ramucirumab with other therapeutics active in HCC such as sorafenib or nivolumab produces enhanced therapeutic response. Considering the inhibitory activity of lenvatinib in multiple RTKs including VEGFR2 and FGFR4, it is possible that subsets of patients may particularly benefit from lenvatinib, such as those with elevated serum AFP and FGF19 + HCC (as demonstrated in the study using ramucirumab and BLU-554, respectively).

## 6. Biliary Tract Carcinoma 

The prognosis of patients with biliary tract carcinoma (BTC) is generally poor, and effective treatment is desperately needed to improve treatment response and survival [19]. For patients who have localized BTC being surgically resected, tumors tend to recur both locally and as distant metastasis. Even when BTC is completely surgically removed (R0 resection), tumor recurrence may occur and the current standard of care is observation [20]. However, a meta-analysis indicated that adjuvant chemotherapy provides a benefit of overall survival in patients with resected BTC [21]. Moreover, for patients with radically resected extrahepatic cholangiocarcinoma and gallbladder carcinoma, adjuvant capecitabine and gemcitabine followed by capecitabine concurrent with radiation therapy was efficacious and well tolerated [22]. Recently, the results of a clinical trial that investigated the role of adjuvant capecitabine in resected BTC have been reported. Besides, for advanced or metastatic BTC, gemcitabine and cisplatin are the standard first-line treatment though with limited survival benefit [20]. The potential value of combining *nab*-paclitaxel with gemcitabine and cisplatin as first-line treatment of advanced BTC has been examined in a clinical study.

### 6.1. Adjuvant Capecitabine in Resected BTC

In a phase III clinical trial, the survival benefit of adjuvant capecitabine versus observation in resected BTC was investigated. In this study, 447 patients with surgical removed BTC (R0 resection) were enrolled. BTC in these patients include intrahepatic (19%), hilar (28%), extra-hepatic (35%) cholangiocarcinoma, and gallbladder carcinoma (18%) [23]. In the treatment arm, the subjects received adjuvant capecitabine 1250 mg/m^2^ on day 1 through day 14 of every 21-day cycle for a total of 8 cycles. The subjects in the control arm were observed with no treatment following surgery. Adjuvant capecitabine significantly prolonged overall survival and recurrence-free survival in resected BTC (Table 9). Results of this study suggest adjuvant capecitabine as the new standard of care for resected (R0) BTC.

### 6.2. Combination of nab-Paclitaxel, Gemcitabine, and Cisplatin in Advanced BTC

For patients with locally advanced unresectable or metastatic BTC, palliative systemic chemotherapy using gemcitabine and cisplatin is the standard of care [20]. The clinical efficacy of the combination of nab-paclitaxel, gemcitabine, and cisplatin as 1st-line treatment of advanced BTC was investigated. In a phase II trial, a single arm of 51 patients were enrolled for treatment using nab-paclitaxel in combination with gemcitabine and cisplatin [24]. Results of this study suggest that the combination of nab-paclitaxel with gemcitabine and cisplatin may provide additional survival benefit as compared with historical control using gemcitabine and cisplatin (Table 10). A phase III clinical trial is indicated to test this hypothesis.

While the phase III study results support the use of adjuvant capecitabine as the new standard of care for resected (R0) BTC, the optimal adjuvant therapy for patients with R1 or R2 resected BTC, as well as tumors with lymph nodes and/or resection margins involved by invasive carcinoma, remains to be determined. Whether the combination of nab-paclitaxel with gemcitabine and cisplatin provides superior survival benefit as compared to gemcitabine/cisplatin will need to be directly compared in a randomized, placebo-controlled clinical study. While the combination of gemcitabine and cisplatin is the current standard treatment for advanced or metastatic BTC, the roles of targeted therapy and immunotherapy in BTC remain to be explored.

## 7. Colorectal Carcinoma

Systemic chemotherapy and targeted therapy have been used as the standard of care for patients with colorectal carcinoma (CRC), as neoadjuvant therapy, adjuvant therapy, and palliative treatment. In this section, results of recent clinical studies investigating (i) immunotherapy (nivolumab), (ii) duration of adjuvant chemotherapy (oxaliplatin and either 5-fluorouracil or capecitabine), (iii) targeted therapy (irinotecan and cetuximab ± vemurafinib), and (iv) palliative chemotherapy (trifluridine/tipiracil and bevacizumab) are presented and discussed.

### 7.1. Nivolumab

Metastatic CRC with DNA mismatch repair-deficient (dMMR) or microsatellite instability-high (MSI-H) displays high levels of tumor-associated neoantigens and tumor-infiltrating lymphocytes. These pathological features suggest anti-tumor response as observed with anti-PD-1 antibodies in other types of tumors. In a multicenter, open-label, single-arm, phase II clinical trial, the therapeutic efficacy of nivolumab was investigated in patients with metastatic CRC who had progressed or been intolerant of ≥1 line of treatment (CheckMate 142, NCT02060188). Patients received nivolumab 3 mg/kg iv every 2 weeks until tumor progression, intolerable toxicity, death, or withdrawal from study. At a median follow-up of 12 months, 23 of 74 enrolled patients showed objective tumor response, and 51 patients had disease control for ≥12 weeks [25]. Results of this clinical study have led to U.S. FDA approval of nivolumab for treatment of patients with metastatic CRC with MSI-H or dMMR previously treated with chemotherapy (fluoropyrimidine and either oxaliplatin or irinotecan).

### 7.2. Adjuvant FOLFOX or CAPOX: 3 Months vs. 6 Months

Adjuvant chemotherapy using oxaliplatin in combination with either 5-fluorouracil or capecitabine (FOLFOX and CAPOX, respectively) for 6 months has been the standard of care for patients following surgical resection of stage III or high-risk stage II CRC. A major drawback of this treatment is that, oxaliplatin-induced neurotoxicity tends to accumulate over time and possibly become permanent.

To determine if adjuvant chemotherapy (either FOLFOX or CAPOX) for 3 months was as effective as 6 months, a global prospective study known as the International Duration Evaluation of Adjuvant therapy (IDEA) was planned. The IDEA study combined data from six concurrent, phase III clinical trials conducted in twelve countries in North America, Europe, and Asia. More than 12,800 patients were enrolled and followed for a median time of 39 months [26]. Results of the IDEA study showed that the rate of disease-free survival with a 3-month course of adjuvant chemotherapy was slightly lower as compared to the standard 6-month course, 74.6% vs. 75.5%, respectively. Moreover, in a subset of patients with low-risk colon cancer (60% of patients in the IDEA study, pT1-T3, pN1), the rates of recurrence-free survival in patients receiving a 3-month course vs a 6-month course were 83.1% and 83.3%, respectively. Besides, patients who received a 6-month course of chemotherapy experienced more side effects including fatigue, diarrhea, and neuropathy than those receiving a 3-month course. In particular, the rates of grade ≥2 chemotherapy-induced neuropathy for patients who received 6 months vs. 3 months of chemotherapy were 45% vs. 15% with FOLFOX, and 48% vs. 17% with CAPOX.

One of those trials in the IDEA collaboration is a non-inferiority, randomized (1:1) clinical study aimed to evaluate if 3 months of adjuvant FOLFOX or CAPOX as effective as 6 months’ adjuvant chemotherapy in stage III or high-risk stage II CRC (SCOT study). In this phase III, open-label, multi-center study, 6088 patients with either stage III or high-risk stage II CRC were enrolled, 32.5% of patients received FOLFOX and 67.5% CAPOX [27]. Results of this study show that 3 months of adjuvant chemotherapy is not inferior to 6 months of treatment (Table 11).

Based on these data, for low-risk disease (T1-3, N1), the recommendation for the duration of adjuvant chemotherapy is 3 months; for high-risk disease (T4 or N2 tumors), the use of shorter course of adjuvant chemotherapy should be tailored to the individual patient.

### 7.3. Irinotecan and Cetuximab ± Vemurafinib

Patients with metastatic CRC that carry the *BRAF*^V600^ mutation tend to respond poorly to standard chemotherapy and/or the BRAF inhibitor vemurafenib. In vitro blockade of *BRAF*^V600^ mutation by vemurafenib has been shown to up-regulate epidermal growth factor receptor (EGFR); a combination of the EGFR inhibitor cetuximab and irinotecan can block the EGFR-mediated signaling events. A randomized clinical study aimed to investigate a combination of irinotecan and cetuximab with or without vemurafenib in *BRAF*^V600^-mutated metastatic CRC (SWOG S1406). In this study, 106 patients with *BRAF*^V600^ mutated and RAS wild-type metastatic CRC were enrolled. The patients had received 1 or 2 prior chemotherapy, 39% of the patients received prior irinotecan, and none had prior anti-EGFR antibodies [28]. Addition of vemurafenib to irinotecan and cetuximab improved anti-tumor response and patient survival (Table 12). Results of this study suggest the combination of vemurafenib with cetuximab and irinotecan as a potential treatment option for patients with *BRAF*^V600^ mutated and RAS wild-type metastatic CRC.

### 7.4. Trifluridine/Tipiracil and Bevacizumab

Previous study has demonstrated a significant overall survival benefit of TAS-102, a combination of trifluridine and tipiracil, in patients with refractory metastatic CRC. A phase I/II, open-label, single-arm, multi-center clinical trial aimed to investigate TAS-102 in combination with bevacizumab in patients with metastatic CRC that had been refractory or intolerant to chemotherapy, anti-VEGF therapy, and anti-EGFR therapy, but had not received regorafenib. In this study, 25 patients were enrolled; 6 patients in phase 1 (dose-escalation) and 19 in phase II. The recommended phase II dose was determined for TAS-102 (35 mg/m^2^ orally twice daily on days 1 to 5 and days 8 to 12 of every 28-day cycle) in combination with bevacizumab (5 mg/kg iv over 30 min every 2 weeks). Results of this study demonstrate a PFS of 42.9% (80% confidence interval 27.8 to 59.0) at 16 weeks, with myelosuppression as the most common grade 3 or 4 adverse event. The mutational statuses of RAS, TP53, APC, and PIK3CA were not associated with survival. These data suggest the combination of TAS-102 with bevacizumab as a new potential treatment option for patients with refractory metastatic CRC [29].

For patients with advanced or metastatic CRC, new therapeutic tools have become available, and new potential treatment options are underway. Since FDA’s approval of nivolumab for treatment of patients with previously treated metastatic CRC with MSI-H or dMMR, molecular analysis for MSI and MMR should be routinely conducted. Any improvement of survival benefit by combination of nivolumab with other therapeutic agents is under active investigation. A 3-month course of adjuvant FOLFOX or CAPOX is expected to be the standard of care for low-risk CRC in patients who will likely benefit from reduced chemotherapy-induced toxicity, particularly peripheral neuropathy. The combination of vemurafenib with cetuximab and irinotecan has the potential of becoming a treatment option for patients with metastatic CRC that carry *BRAF*^V600^ mutation and wild-type RAS. The combination of trifluridine/tipiracil and bevacizumab appears an attractive option for patients with refractory metastatic CRC. A phase III clinical study to compare the efficacy and safety of this combination with the current standard treatment, and even addition of oxaliplatin or irinotecan to the combination may be considered in future investigation.

## 8. Conclusions and Future Perspectives

In this article, the presentations on the new development in systemic treatment of various malignant diseases in the digestive system at the Multi-Disciplinary Patient Care in Gastrointestinal Oncology Conference on 29 September 2017 are summarized. These include highlights of the recent clinical trials that investigated chemotherapy, targeted therapy, and immunotherapy in the malignant diseases of various digestive organs. Results of some of the clinical studies have led to U.S. FDA approval of nivolumab in HCC and CRC with dMMR or MSI-H, and pembrolizumab in GC/GEJC expressing PD-L1. The recent FDA’s approval of pembrolizumab for treatment of solid tumors, including those in the digestive organs, that display dMMR also needs to be mentioned [30]. Peri-operative FLOT for resectable GC and GEJC, adjuvant capecitabine for resected (R0) BTC, and 3 months of adjuvant FOLFOX or CAPOX for low-risk CRC are expected to become new standard of care. Various targeted therapeutic agents, and new combinations of chemotherapy and targeted therapy demonstrate the potential of providing new treatment options for patients with cancers of the digestive system.

## Figures and Tables

**Table 1:** Results of a phase II clinical study to investigate pegylated hyaluronidase in combination with nab-paclitaxel and gemcitabine in metastatic pancreatic carcinoma.

Outcomes	PEGPH20 + nab-Paclitaxel + Gemcitabine	nab-Paclitaxel + Gemcitabine
PFS (months) All patients	6.0 HR 0.73, * *P* < 0.05	5.3
PFS (months) HA-High (35%)	9.2 HR 0.51, * *P* < 0.048	5.2
OS (months) HA-High (35%)	11.5	8.5

HA: hyaluronan. HR: hazard ratio. OS: overall survival. PEGPH20: pegylated hyaluronidase. PFS: progression-free survival. * *P* indicates statistical significance.

**Table 2:** Results of a phase I/II clinical study to investigate napabucasin in combination with *nab*-paclitaxel and gemcitabine in metastatic pancreatic adenocarcinoma.

Efficacy Endpoints	Outcome
Complete Response	1 (1.8%)
Partial Response	26 (47.3%)
Stable Disease	24 (43.6%)
Overall Response Rate	55%
Disease Control Rate	93%
Disease Progression	3 (on treatment), 1 after off treatment due to toxicity
Progression-Free Survival	7.1 months
1-Year Overall Survival Rate	56%

**Table 3:** Results of peri-operative FLOT in patients with resectable gastric or gastroesophageal junction carcinoma.

Regimens	PFS (Months)	OS (Months)	PD during or after Pre-Op	pT0/T1	R0 Resection
FLOT	30 HR 0.75, * *P* = 0.004	50 HR 0.77, * *P* = 0.012	1% * *P* = 0.001	25% * *P* = 0.001	84% * *P* = 0.001
ECF or ECX	18	35	5%	15%	77%

ECF: epirubicin, cisplatin, 5-fluorouracil. ECX: epirubicin, cisplatin, capecitabine. FLOT: 5-fluorouracil, leucovorin, oxaliplatin, docetaxel. OS: overall survival. PD: progression of disease. PFS: progression-free survival. * *P* indicates statistical significance.

**Table 4:** Results of a phase II clinical study to investigate nivolumab and ipilimumab in metastatic gastric or gastroesophageal junction carcinoma.

Outcomes	Nivolumab (3 mg/kg) Every 2 Weeks	Nivolumab (1 mg/kg) + Ipilimumab (3 mg/kg) every 3 Weeks	Nivolumab (3 mg/kg) + Ipilimumab (1 mg/kg) Every 3 Weeks
ORR (%)	12	24	8
ORR (%) PD-L1 ≥ 1%	19	40	23
ORR (%) PD-L1 ≤ 1%	12	22	0
OS (months)	6.2	6.9	4.8

ORR: overall response rate. OS: overall survival.

**Table 5:** Results of a clinical study to investigate nivolumab in advanced hepatocellular carcinoma.

Outcomes	Sorafenib-Naïve (*n* = 80)	Sorafenib-Treated (*n* = 182)
Overall response rate	24%	19%
Complete response	1%	1%
Partial response	19%	13%
Stable disease	34%	41%
Disease control rate	63%	56%

**Table 6:** Results of a phase III clinical study to investigate ramucirumab in advanced hepatocellular carcinoma.

Outcomes	Ramucirumab vs. Placebo	Ramucirumab vs. Placebo (AFP ≥ 400 ng/mL)
HR (Child-Pugh 5)	0.80 (*P* = 0.06)	0.61 (* *P* = 0.01)
HR (Child-Pugh 6)	0.96 (*P* = 0.76)	0.64 (* *P* = 0.04)
HR (Child-Pugh 7 or 8)	1.00 (*P* > 0.99)	0.67 (*P* = 0.28)

AFP: Alpha fetoprotein; HR: Hazard Ratio. * *P* indicates statistical significance.

**Table 7:** Results of a phase III clinical trial to investigate lenvatinib vs sorafenib in unresectable hepatocellular carcinoma.

Outcomes	Lenvatinib	Sorafenib
Median OS (months)	13.6 HR 0.92; NS	12.3
Median PFS (months)	7.4 HR 0.66; * *P* < 0.00001	3.7
Median TTP (months)	8.9 HR 0.63; * *P* < 0.00001	3.7
ORR (%)	24 * *P* < 0.00001	9

HR: hazard ratio. NS, not statistically significant. ORR: overall response rate. OS: overall survival. PFS: progression-free survival. TTP: time to tumor progression. * *P* indicates statistical significance.

**Table 8:** Results of a phase I clinical trial to investigate BLU-544 in pretreated advanced hepatocellular carcinoma.

Outcomes	Result
Complete Response	1 patient
Partial Response	5 patients
Stable Disease	20 patients
Overall Response Rate	16%
Disease Control Rate	68%

**Table 9:** Results of a phase III clinical trial to investigate adjuvant capecitabine in resected (R0) biliary tract carcinoma.

Regimen	Capecitabine	Observation
Overall survival	51 months HR 0.75; * *P* = 0.028	36 months
Recurrence-free survival	25 months	18 months

HR: Hazard ratio. * *P* indicates statistical significance.

**Table 10:** Results of a phase II clinical trial to investigate nab-paclitaxel in combination with gemcitabine and cisplatin in advanced biliary tract carcinoma.

Regimen	Progression-Free Survival	Overall Survival	1-Year Survival Rate
nab-Paclitaxel + Gemcitabine + Cisplatin	11.4 months	>20 months (estimated)	66.7%
Gemcitabine + Cisplatin	8.0 months (Historical control)	11.7 months (Historical control)	-

**Table 11:** Results of a phase III clinical study to investigate the efficacy of 3 months vs. 6 months of adjuvant chemotherapy in surgically resected stage III or high-risk stage II colorectal carcinoma.

Outcome	3 Months of CAPOX or FOLFOX	6 Months of CAPOX or FOLFOX
DFS Events	734	735
3-Year DFS Rate (%)	76.8 HR 1.008 (95% CI 0.910–1.117) Non-inferiority, * *P* = 0.014	77.4

CAPOX: capecitabine and oxaliplatin. CI: confidence interval. DFS: disease-free survival. FOLFOX: 5-fluorouracil, leucovorin, and oxaliplatin. HR: hazard ratio. * *P* indicates statistical significance.

**Table 12:** Results of a clinical study to investigate the addition of vemurafenib to irinotecan and cetuximab in metastatic colorectal carcinoma with *BRAF*^V600^ mutation and wild-type RAS.

Regimen	Vemurafenib + Irinotecan and Cetuximab	Irinotecan and Cetuximab
PFS (months)	4.4 (5.7 if no prior irinotecan)	2.0 (1.9 if no prior irinotecan)
RR (%)	16 ^#^ *P* = 0.08	4
DCR (%)	67%	22

DCR: disease control rate. PFS: progression-free survival. RR: response rate. ^#^ *P* indicates a trend for statistical significance.
